# Effective targeting of microglial P2X7 following intracerebroventricular delivery of nanobodies and nanobody-encoding AAVs

**DOI:** 10.3389/fphar.2022.1029236

**Published:** 2022-10-10

**Authors:** Carolina Pinto-Espinoza, Charlotte Guillou, Björn Rissiek, Maximilian Wilmes, Ehsan Javidi, Nicole Schwarz, Marten Junge, Friedrich Haag, Nastassia Liaukouskaya, Nicola Wanner, Annette Nicke, Catelijne Stortelers, Yossan-Var Tan, Sahil Adriouch, Tim Magnus, Friedrich Koch-Nolte

**Affiliations:** ^1^ Institute of Immunology, University Medical Center Hamburg-Eppendorf, Hamburg, Germany; ^2^ Department of Neurology, University Medical Center Hamburg-Eppendorf, Hamburg, Germany; ^3^ Normandie Univ, UNIROUEN, INSERM U1234, Pathophysiology, Autoimmunity and Immunotherapy (PanTHER), Rouen, France; ^4^ MSH- Medical School Hamburg- Dep. Anatomy, Hamburg, Germany; ^5^ Department of Nephrology, University Medical Centre Hamburg-Eppendorf, Hamburg, Germany; ^6^ Walther Straub Institute of Pharmacology and Toxicology, Faculty of Medicine, LMU Munich, Munich, Germany; ^7^ Ablynx-Sanofi, Ghent, Belgium

**Keywords:** P2X7 nanobodies, receptor occupancy, functional blockade, intracerebroventricular delivery, AAV-mediated delivery

## Abstract

The P2X7 ion channel is a key sensor for extracellular ATP and a key trigger of sterile inflammation. Intravenous injection of nanobodies that block P2X7 has shown to be beneficial in mouse models of systemic inflammation. P2X7 has also emerged as an attractive therapeutic target for inflammatory brain diseases. However, little is known about the ability of nanobodies to cross the BBB. Here we evaluated the ability of P2X7-specific nanobodies to reach and to block P2X7 on microglia following intravenous or intracerebral administration. For this study, we reformatted and sequence-optimized P2X7 nanobodies for higher stability and elevated isoelectric point. Following injection of nanobodies or nanobody-encoding adeno-associated viral vectors (AAV), we monitored the occupancy and blockade of microglial P2X7 *in vivo* using *ex vivo* flow cytometry. Our results show that P2X7 on microglia was within minutes completely occupied and blocked by intracerebroventricularly injected nanobodies, even at low doses. In contrast, very high doses were required to achieve similar effects when injected intravenously. The endogenous production of P2X7-antagonistic nanobodies following intracerebral or intramuscular injection of nanobody-encoding AAVs resulted in a long-term occupancy and blockade of P2X7 on microglia. Our results provide new insights into the conditions for the delivery of nanobodies to microglial P2X7 and point to AAV-mediated delivery of P2X7 nanobodies as a promising strategy for the treatment of sterile brain inflammation.

## 1 Introduction

P2X7 is a ligand-gated non-selective cation channel that structurally assembles as a homomeric trimer (Nicke, 2008). P2X7 is expressed on the cell surface of various leukocytes, particularly at high levels on macrophages, microglia (Ferrari et al., 1996; He et al., 2017; Metzger et al., 2017) and resident T cells of the kidney (Stark et al., 2018; Borges da Silva et al., 2020). The physiological ligand of P2X7 is extracellular ATP. During inflammation, stressed and injured cells release large amounts of ATP that act as a danger associated molecular pattern (DAMP) (Choi et al., 2007; Falzoni et al., 2013). In LPS-primed microglia and monocytes, gating of P2X7 by ATP leads to the influx of Na^+^ and Ca^2+^ and the efflux of K^+^ (Choi and Kim, 1996; Egan and Khakh, 2004). Intracellular K^+^ depletion triggers the assembly of the NLRP3 inflammasome, a multiprotein complex that drives caspase-1-mediated maturation and release of leaderless IL-1β, a key cytokine of the inflammatory response (Perregaux and Gabel, 1994; Ferrari et al., 1997; Sanz and Di Virgilio, 2000). Caspase-1 also cleaves Gasdermin D to generate free N-terminal domains which oligomerize at the plasma membrane to form pores through which mature IL-1β is released (Shi et al., 2015; Ding et al., 2016; Evavold et al., 2018; Heilig et al., 2018). *In vitro*, ATP-induced pore formation has also been used to monitor P2X7 activation based on the increased membrane permeability to fluorescent molecules as for instance DNA-staining dyes such as DAPI (Yan et al., 2008).

In the CNS, inflammation and ischemia result in the local release of ATP at the site of tissue injury (Melani et al., 2005; Sanz et al., 2009; Grygorowicz et al., 2016). In addition, P2X7 activation on microglia has been implicated in the pathogenesis of experimental autoimmune encephalomyelitis (EAE) (Matute et al., 2007; Grygorowicz and Struzynska, 2019), Alzheimer disease (AD) (McLarnon et al., 2006), and stroke (Franke et al., 2004; Melani et al., 2006; Skaper et al., 2006), which are often associated with an increased release of IL-1β (Boutin et al., 2001; Rampe et al., 2004; Jin et al., 2008; Sanz et al., 2009; Maysami et al., 2016; Grygorowicz and Struzynska, 2019).

Pharmacological antagonists of P2X7 have been proposed as potential therapeutic tools to control inflammation in the brain. Because most of them have shown lack of specificity, low potency, diverse pharmacodynamics, and conversion into ineffective or toxic metabolites (Bartlett et al., 2014; Bhattacharya and Biber, 2016; Andrejew et al., 2020), only the BBB-penetrant JNJ-54175446 has entered into phase II clinical trials for inflammation (Recourt et al., 2020). Antibodies are highly desirable therapeutic molecules as they offer high specificity, safety, uniform pharmacodynamics, and low toxicity. Nanobodies are antibody fragments consisting of a single variable domain (VHH) of a heavy chain antibody. Nanobodies are highly soluble and with a small size (∼12 kDa), they bind to their target with high specificity, similar to conventional antibodies (Hamers-Casterman et al., 1993; Nguyen et al., 2000; Conrath et al., 2005; Wesolowski et al., 2009). We have previously generated highly specific P2X7-antagonistic nanobodies that dampened inflammation in mouse models of allergic contact dermatitis and experimental glomerulonephritis (Danquah et al., 2016). However, similar to other biologics, the therapeutic application of P2X7-antagonistic nanobodies in CNS inflammation encounters the BBB as physical obstacle which restricts the passage of molecules with a MW > 500 Da (Fischer et al., 1998; Pardridge, 2005).

Our goal was to evaluate the CNS delivery of two potent P2X7-blocking nanobodies, that differ in their isoelectric point (pI), using different routes of administration. Nanobody 13A7 (pI 5.9) specifically blocks ATP-induced activation of mouse P2X7 while nanobody 1c81^s^ (pI 9.7) blocks both mouse and human P2X7, making it of interest for translational studies. We developed a sensitive cell-based assay to determine the level of P2X7 occupancy on brain cells (microglia) in contrast to cells in peripheral tissue (resident T cells of the kidney, T_RMs_). We chose these cell types because of their known high levels of P2X7 on the cell surface (Metzger et al., 2017; Stark et al., 2018). We used this assay to evaluate the pharmacodynamics of P2X7 nanobodies after intravenous or intracerebral injections, as well as after intramuscular delivery of nanobody-encoding AAV1. The results demonstrate that an effective targeting of P2X7 on microglia *in vivo* can be readily achieved by intracerebroventricular injection of P2X7 antagonistic nanobodies, even at low doses, but very high doses are required when injected intravenously. Furthermore, the endogenous production of P2X7-antagonistic nanobodies 120 days after transduction of muscle cells with nanobody-encoding AAVs resulted in a high, albeit not complete, occupancy and blockade of P2X7 on microglia, whereas transduction of brain cells with nanobody-encoding AAVs resulted, 28 days later, in a complete blockade of P2X7 on microglia.

## 2 Materials and methods

### 2.1 Antibodies and reagents

The following fluorochrome conjugated monoclonal antibodies (mAbs) were used for flow cytometry: anti-CD45 (30-F11), anti-CD4 (GK1.5), anti-CD8 (53-6.7), anti-CD69 (H1.2F3), anti-CD11b (M1/70), anti-mouse IgG1 (RMG1-1), and purified anti-CD16/CD32, all purchased from Biolegend. Purified anti-VHH mouse IgG1 antibodies mAbH0077 and mAbH0074 were received from Ablynx and mAbs specific for mouse P2X1, P2X4, P2X7 were produced in house (Moller et al., 2007). Dead-cell staining was performed using DAPI and/or the AlexaFluor700 LIVE/DEAD Fixable Read Dead Stain Kit (Biolegend). For immunofluorescence microscopy the following fluorochrome conjugated reagents were used: isolectin purchased from Thermofisher, anti-GFP B-2 mAb (sc-9996) from Santa Cruz, anti-ALFA tag nanobody (1G5) from Nano-tag technologies and purified anti-ALFA tag nanobody fused to a rabbit IgG produced in house. DNA staining was performed with Hoechst 33342 (Thermofisher). ATP disodium salt was purchased from Sigma.

### 2.2 Construction and production of dimeric half-life extended nanobodies

P2X7-specific nanobodies 13A7, 14D5 and a sequence optimized variant of 1c81 with higher stability and elevated isoelectric point were engineered into bivalent half-life extended nanobodies, or bivalent Fc-fused format, as previously described (Danquah et al., 2016). Nanobody-constructs were subcloned into the pCSE2.5 expression vector either carrying at the C-terminus a tandem SNB tag (Schaffer et al., 2010) or carrying at the N-terminus an ALFA-tag (Gotzke et al., 2019). All nanobody constructs were produced as secretory proteins in transiently transfected HEK-6E cells cultivated in serum-free medium (cells provided by Ives Durocher, Montreal, Canada). After 6 days, cell-supernatants were harvested and clarified by centrifugation. Nanobodies were purified from the supernatants by affinity chromatography using protein A columns (GE Healthcare) and formulated at high concentrations (15–50 mg/ml) in an in-house low aggregation buffer (5.8 mg/ml sodium phosphate monobasic, monohydrate; 1.2 mg/ml sodium phosphate dibasic, anhydrous; 60 mg/ml trehalose; 0.4 mg/ml Tween^®^; pH 6.5). Integrity and purity were confirmed by SDS-PAGE and Coomassie brilliant blue staining.

### 2.3 Cell-based P2X7 binding assay

HEK 293 cells were transiently transfected with expression plasmids encoding the indicated proteins (eGFP, P2X1, P2X4, P2X7) using Lipofectamine 2000 (Life Technologies) according to the manufacturer’s recommendations. Cells were then sequentially incubated with the indicated nanobodies, mAbH0077 and BV421-conjugated anti-mouse IgG1 antibody. For the expression controls, cells were stained with the indicated AF647-conjugated specific mAbs (anti-mouse P2X1, P2X4, P2X7, in house). Cells were analyzed by flow cytometry (BD-FACS-Celesta).

### 2.4 Real-time and endpoint calcium influx and DAPI uptake assays

For the real time experiments, primary mixed glial co-cultures were loaded with 2 μM Fluo-4 (Invitrogen) for 15 min at 37°C, washed and resuspended in PBS supplemented with Ca^2+^ and Mg^2+^. Cells were incubated with 1 μg of the indicated nanobodies for 20 min and after addition of DAPI, cells were analyzed by flow cytometry (BD-FACS-Canto). An infrared lamp was used to maintain a constant temperature during sample measurement. After equilibration, 0.5 mM ATP (Sigma) was added at the indicated time point (Veltkamp et al., 2022). Alternatively, Fluo-4 loaded HEK cells stably expressing mouse P2X7 were incubated with the indicated serially diluted nanobodies and stimulated with 1.5 mM ATP for 15 min. Intracellular Ca^2+^ was measured by fluorimetry on a plate reader heated at 37°C (Victor3, Perkin Elmer). For endpoint DAPI uptake assay, brain cells from nanobody-injected mice were stimulated with 0.5 mM ATP in RPMI containing DAPI, in presence or absence of 0.5 μg of the same nanobody. Cells were washed in ice cold FACS buffer and CD11b^+^ CD45^+^ microglia were analyzed by flow cytometry.

### 2.5 IL-1β release assays

Mixed glial primary co-cultures were incubated at 37°C sequentially with LPS (1 μg/ml) for 4 h and with serial dilutions of the respective nanobodies for an additional 30 min. ATP (0.5 mM) was then added to the cells and, after 30 min of stimulation, the cells were centrifuged. IL-1β levels were determined in the supernatants by ELISA (Biolegend).

### 2.6 Animals

Wild-type, P2X7^−/−^ (Labasi et al., 2002) and BAC transgenic P2X7-eGFP (line 17) (Kaczmarek-Hajek et al., 2018) mice in C57BL/6 background (8–12 weeks old) were bred in the animal facility of the University Medical Center Hamburg-Eppendorf. Animals had free access to water and standard animal chow. All animal experiments and experiments involving tissue derived from animals were performed with approval of the responsible regulatory committee (Hamburger Behörde für Justiz und Verbraucherschutz, G12/130, N006/2018, 021/18, and French Ministry of Higher Education, Research, and Innovation, APAFIS #27816). All methods were performed in accordance with the relevant guidelines and regulations.

### 2.7 Systemic injections of nanobodies and fluorochrome-conjugated CD45-specific mAb

Purified nanobodies were adjusted to the indicated concentrations in a sodium-phosphate buffer containing trehalose (60 mg/ml) and Tween^®^ (0.4 mg/ml) and injected at the indicated doses either i.v. (100 µl) into the tail vein or i.c.v. (2 µl). PerCP-conjugated anti-CD45 mAb was adjusted to a concentration of 10 μg/ml, and 100 µl (1 µg) were injected into the tail vein 2–3 min before sacrifice. Mice were deeply anesthetized by isoflurane inhalation and then perfused transcardially with PBS-heparin (10 U/ml) for 4 min using a peristaltic pump at a rate of 8 ml/min. Shortly before perfusion, blood was drawn by terminal cardiac puncture. Nanobody encoding-AAV1 viral vectors were produced by Virovek, Hayward, United States (Gonde et al., 2021). For muscle transduction, mice were anesthetized and 10^11^ viral genomes in 50 μl of PBS were injected per mouse in the gastrocnemius muscle located in the hindlimb. For CNS cell transduction, as above, 10^11^ viral genomes of nanobody-encoding AAV1 viral vectors were administered either i.c.v. (4 μl) or intracisterna magna (i.c.m.) (20 μl) per mouse.

### 2.8 Preparation of microglia-astrocyte co-cultures

Neonatal C57BL6 mice were sacrificed by decapitation. Brains were dissected and the olfactory bulb, cerebellum and meninges were removed under the microscope. A cell suspension was prepared by mincing brain tissue in Hank’s balanced salt solution. Cell pellets were collected and treated with 0.5 mg/ml papain (Sigma) and 0.01 mg/ml deoxyribonuclease type I (Sigma) in Hank’s balanced salt solution, for 30 min at 37°C. Cells were then resuspended with BME medium supplemented with 10% FCS and filtered through a 70 μm cell strainer (EASY strainer, GBO). Cell pellets were collected, resuspended in 10% FCS BME medium and cultured in T-75 flasks (Sigma) for 21 days at 37°C with 5% CO_2_.

### 2.9 Preparation of primary brain microglia, kidney leukocytes and splenocytes

After removal of the olfactory bulb and cerebellum, brains were cut into small 1–5 mm pieces in DMEM containing 1 mg/ml collagenase A (Roche) and 0.01 mg/ml deoxyribonuclease type I (Sigma) and incubated for 30 min at 37°C. Tissue fragments were dissociated using a 40 μm cell strainer (EASY strainer, GBO). Kidneys were cut with into small 1–5 mm fragments in DMEM containing 10% fetal calf serum, 0.4 mg/ml collagenase D (Roche) and 0.02 mg/ml deoxyribonuclease type I (Sigma) and dissociated at 37°C for 30 min using a GentleMACS apparatus (Miltenyi Biotec). Cell pellets were gently resuspended and layered onto a 30% (brain cells) or 40% (kidney cells) Percoll gradient (GE healthcare) and bands corresponding to leucocytes were collected. Spleens were minced in DMEM, 10% FCS using a 70 μm cell strainer (EASY strainer, GBO). Cells were washed and red-blood-cells were lysed using ACK erythrocyte lysis buffer (155 mM NH_4_Cl, 10 mM KHCO_3_, 0.1 mM EDTA, pH 7.2). After washing, cells were resuspended in FACS Buffer.

### 2.10 Immunofluorescence microscopy

Frozen brain sections from i.c.v. injected mice (5 µm thick) were air-dried, fixed with methanol for 10 min at −20°C and washed with PBS. Autofluorescence was quenched with 50 mM NH^4^Cl for 7 min at room temperature. After washing, unspecific binding was blocked with 5% BSA in PBS for 45 min at room temperature. For immunostaining, sections were rinsed with PBS and incubated with an anti-ALFA-tag nanobody-rabbit IgG in blocking solution overnight at 4°C. After washing with PBS supplement with Ca^2+^ and Mg^2+^, sections were stained with AF488-conjugated isolectin (1:100) and either a AF555-conjugated anti-rabbit IgG antibody or an AF647-conjugated anti-ALFA-tag nanobody (1:500). Sections were counterstained with Hoechst 33342 in PBS supplement with Ca^2+^ and Mg^2+^ for 45 min at room temperature and mounted with ProLongTM Gold antifade mountant (Invitrogen) on glass slides. Staining was evaluated with a THUNDER Imager 3D microscope (Leica Microsystems, Mannheim. Germany) fitted with a LED8 filter setup and a 4.2 MP sCMOS camera. Images were acquired using 40x (NA: 1.1) and 63x (NA: 1.1) objectives.

### 2.11 Flow cytometry

All incubations with antibodies were carried out at 4°C for 20 min, washing steps and cell-resuspension with FACS Buffer (2% BSA, 1 mM EDTA in PBS). Data was collected with a (BD-FACS-Celesta) system using Diva software, and data was analyzed using the Flowjo software (Tree Star).

### 2.12 Statistical analyses

All statistical analyses were performed with the SPSS package and Graph Pad Prism 5.1 software. All data are expressed as means ± SE. The student t test was used for comparison between two groups. In case of three or more groups, one-way ANOVA was used, followed by a post hoc analysis with Bonferroni’s test for multiple comparisons.

## 3 Results

### 3.1 Engineering and functional characterization of nanobodies

**FIGURE 1:**
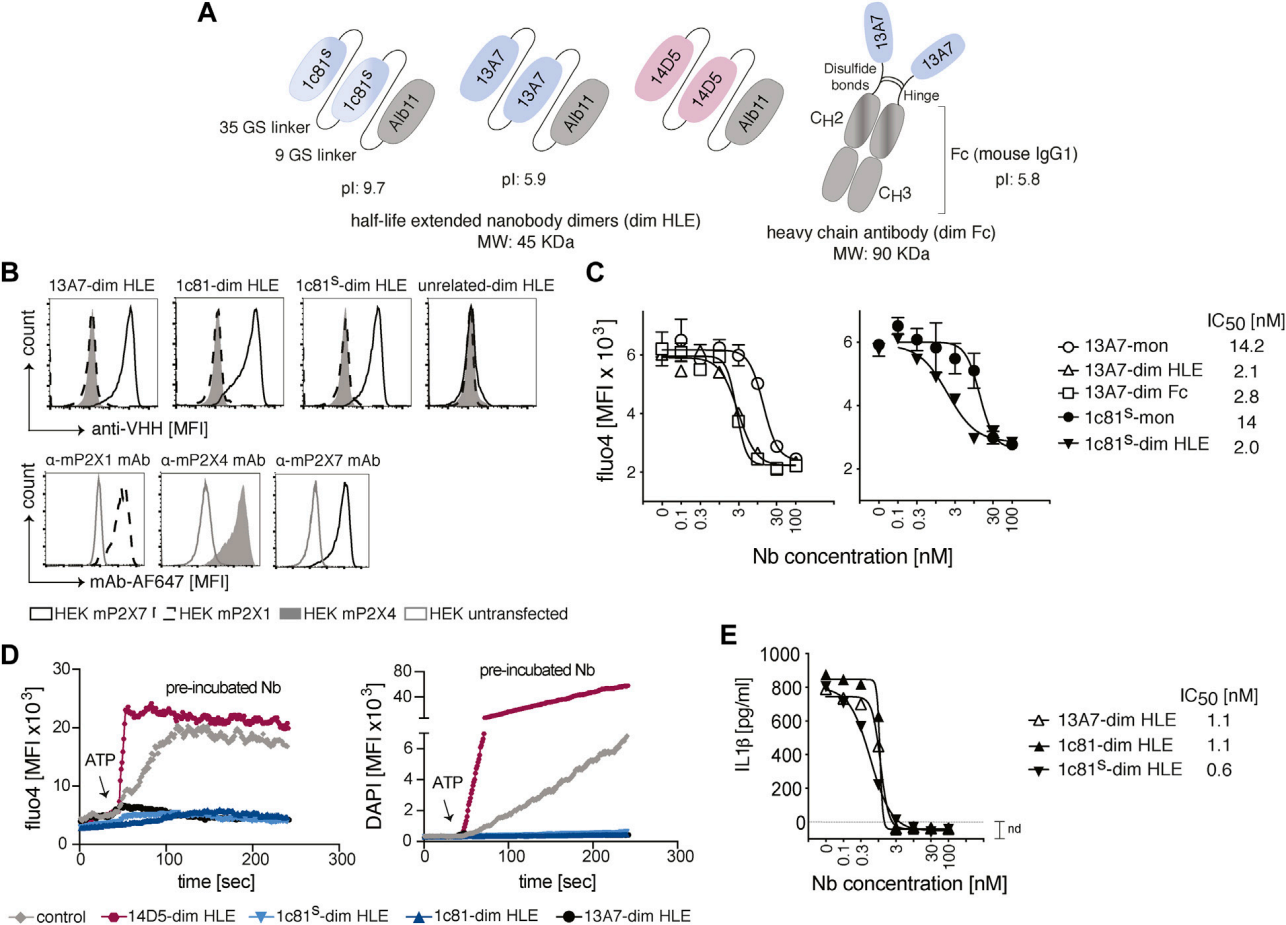
Engineering and functional characterization of P2X7 nanobodies for brain targeting. **(A)** Schematic representation of half-life extended bivalent mouse P2X7-specific nanobodies 1c81^s^, 13A7 and potentiator 14D5 fused to an albumin specific nanobody, Alb11 (dim HLE) or to the hinge and Fc domain of mouse IgG1 (dim Fc). Nanobody dimers are connected through a 35 Gly-Ser linker and fused to Alb11 with a 9 Gly-Ser linker, or are connected to the Fc domain of mouse IgG1 through the hinge region. The predicted molecular weight of the bi-specific trimers is 45 kDa and that of the reconstituted 13A7 mouse IgG1 heavy chain antibody is 90 kDa. The predicted isoelectric point (pI) for dim HLE formats of 1c81^s^ and 13A7 and dim Fc format of 13A7 is shown. **(B)** Binding of P2X7 nanobodies to mouse P2X1, P2X4 or P2X7 on the cell surface of HEK cells co-transfected with GFP and the respective P2X receptor. Bound nanobodies were detected with anti-VHH monoclonal antibody mAbH0077 followed by a BV421-conjugated anti-mouse IgG1 antibody (upper panels). As controls, HEK cells were stained with the indicated AF647-conjugated mAbs (lower panel). Gating of transfected HEK cells was performed on GFP^+^ cells (MFI, mean fluorescence intensity). **(C)** Functionality of P2X7 nanobodies to inhibit the ATP-dependent channel activation in stable mouse P2X7-expressing HEK cells (HEK^P2X7^). HEK^P2X7^ loaded with the Ca^2+^ indicator Fluo-4 were incubated with serial dilutions of monovalent or bivalent HLE nanobodies, and after addition of ATP (1.5 mM), the release of Fluo-4 was monitored over time. The graphs show average Fluo-4 endpoint measurements (n = 3)+/-SD at 20 min. **(D)** Flow cytometric analysis of function of P2X7 nanobodies on primary murine microglia loaded with Fluo-4 in the presence of DAPI. The fluorescence intensity was measured for 4 min following addition of ATP (0.5 mM). The 14D5-dim HLE nanobody enhanced P2X7 function and was included as positive control. **(E)** Functional inhibition of ATP-induced IL-1β release by primary glial cultures in the presence of bivalent HLE nanobodies. The IL-1β concentration was measured by ELISA in the supernatant of cells treated with 1.5 mM ATP for 30 min (n = 3, average+/-SD). All IC_50_ values were calculated with Prism software. Data are representative of two **(B–D)** or three **(E)** independent experiments. Samples in D and E were pooled from two mice.

The P2X7-specific nanobodies (Nb) used in this study were engineered as half-life extended bivalent formats of 13A7 (antagonist) (Danquah et al., 2016), a sequence optimized variant of 1c81 (antagonist) and 14D5 (allosteric enhancer) (Danquah et al., 2016). Dimerization was conceived to increase the binding avidity and thus enhance functional potencies. The half-life extension was achieved either by C-terminal fusion to an albumin-specific nanobody Alb11 (Tijink et al., 2008) (dim HLE), or by fusion to the Fc domain of mouse IgG1 (dim Fc) (Figure 1A). Alb11 binds to the serum albumin, thereby increasing the half-life of the construct, and the Fc-region mediates a longer half-life by binding to the neonatal Fc receptor (FcRn). The molecular weight of the dim HLE format is 45 kDa and that of the dim Fc format is 90 kDa. The increase in size also contributes to half-life extension by reducing renal filtration.

Previous studies reported that anti-GFP nanobodies with an isoelectric point (pI) > 8 show enhanced passage across the BBB, likely following the adsorptive mediated transcytosis mechanism (Li et al., 2012; Li et al., 2016). Therefore, we increased the pI of 1c81 by introducing two basic amino acids in framework regions 1 and 4 (Q3K, Q122K), and fusing to the C-terminus a tandem of the highly basic SNB tag (Schaffer et al., 2010) (Figure 1A).

To verify that the sequence modification did not affect the target binding of 1c81, we analyzed by flow cytometry the binding of 1c81-dim HLE, 1c81^s^-dim HLE and 13A7-dim to HEK cells co-transfected with GFP and expression constructs for either mouse P2X1, P2X4 or P2X7. All engineered nanobodies bound specifically to P2X7 but not to P2X1 and P2X4 (Figure 1B). We also confirmed that the dimerization of these nanobodies increased their blocking potency. For this we monitored the ATP-induced gating of P2X7 revealed by the influx of Ca^2+^ into HEK cells stably expressing mouse P2X7 (HEKP2X7). Fluorometric analyses of Fluo-4 loaded HEKP2X7 showed that bivalent nanobodies blocked the ATP-induced Ca^2+^ influx with 7-fold higher potencies than their monovalent counterparts (Figure 1C). Since extracellular ATP also induces large pore formation in P2X7-expressing cells, we simultaneously monitored DAPI uptake and Ca^2+^ influx in adult primary microglia by real-time flow cytometry. The results showed that 13A7-dim HLE, 1c81-dim HLE and 1c81^s^-dim HLE effectively blocked, during the entire time of measurement, both ATP-induced DAPI uptake and Ca^2+^ influx in microglia. In contrast, 14D5-dim HLE enhanced both ATP-induced induced effects in microglia (Figure 1D).

ATP binding to P2X7 also triggers a K^+^ influx that activates the inflammasome to process caspase-1 (Perregaux and Gabel, 1994). In turn, active caspase-1 cleaves Gasdermin D (GSDMD) and pro-IL-1β into mature IL-1β. Next, the N-terminal GSDMD forms pores in the plasma membrane that mediate the release of IL-1β (Heilig et al., 2018). To assess whether P2X7-antagonistic bivalent HLE nanobodies block IL-1β release by microglia, we measured by ELISA the levels of IL-1β in the supernatant of mixed primary glial cultures primed with LPS and treated with ATP. All evaluated P2X7-antagonistic bivalent HLE nanobodies (13A7-dim HLE, 1c81-dim HLE and 1c81^s^-dim HLE) inhibited the release of IL-1β from microglia in a dose dependent fashion and with high potency (Figure 1E). Taken together, these data demonstrate that ATP-induced Ca^2+^ influx, DAPI uptake and IL-1β release by primary mouse microglia is mediated by P2X7 and that the engineered P2X7-specific nanobodies block with high potency all these effects.

### 3.2 *In vivo* binding of P2X7 bivalent HLE nanobodies to immune cells in the brain and in the kidney

**FIGURE 2:**
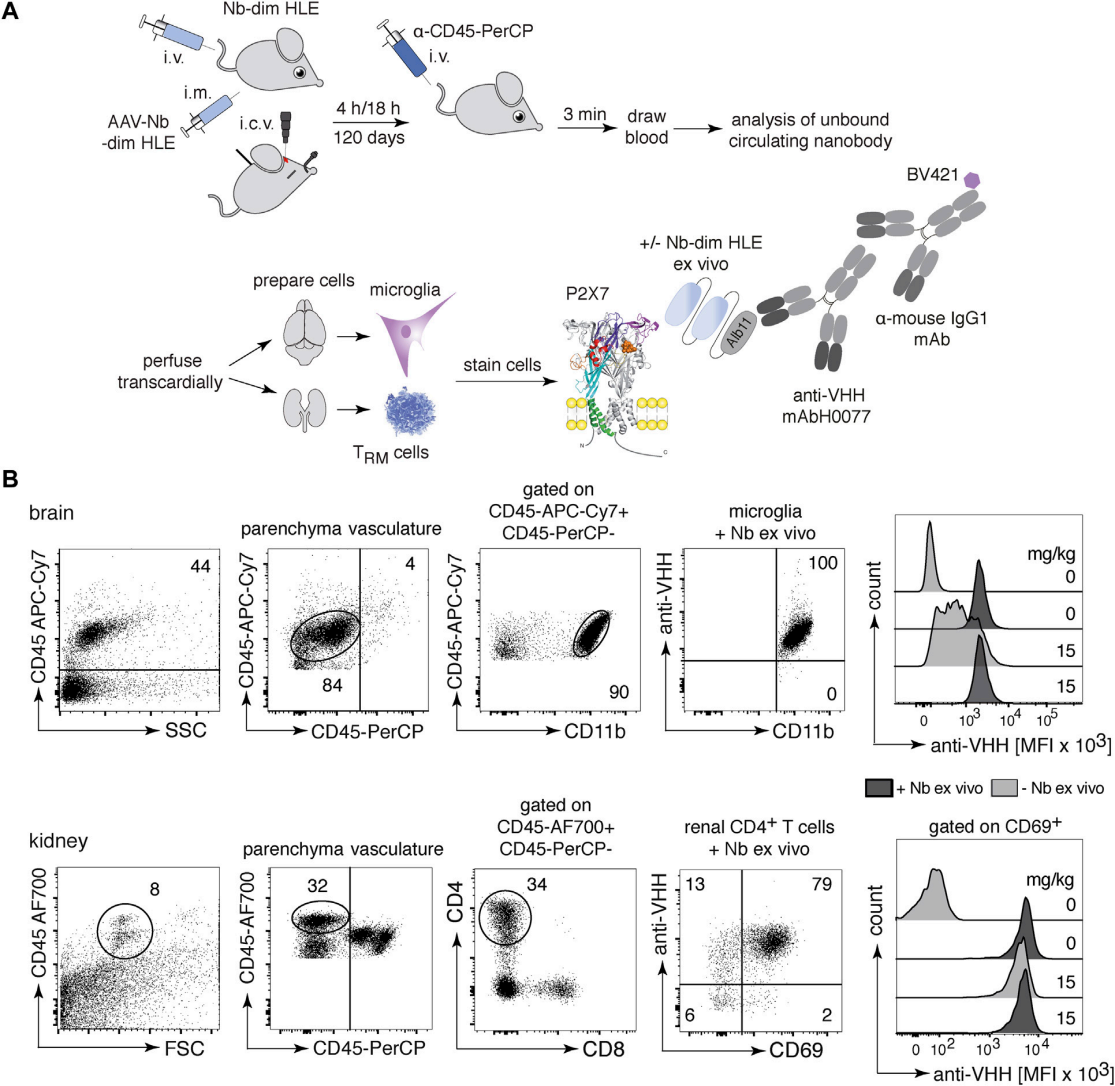
*In vivo* occupancy of cell surface P2X7 by injected bivalent HLE nanobodies. **(A)** The scheme illustrates the experimental set-up to determine the level of target occupancy of P2X7 in different tissues using different routes of administration of P2X7-specific nanobodies. Bivalent HLE nanobodies were injected either i.v. or i.c.v. into mice and sacrificed after 4 h or 18 h. In addition, a single i.m. injection of nanobody-encoding AAV was performed for endogenous production of nanobodies, and mice were sacrificed after 120 days. Prior to sacrifice, a CD45-specific mAb was injected i.v. to stain vascular leukocytes. A blood sample was obtained to determine the level of unbound nanobodies in serum and vascular nanobodies were removed by transcardial perfusion. Brain and kidney cell suspensions were analyzed for cell surface-bound nanobodies by detection with anti-HHV mAbH0077 followed by BV421-conjugated mouse IgG1-specific mAb. As control, cells were incubated *ex vivo* with a saturated dose of the same nanobody (+Nb-dim HLE *ex vivo*) to assess the maximal level of P2X7 occupancy. **(B)** Parenchymal brain cells (top) were detected with a second CD45-specific mAb and microglia were identified as the parenchymal CD45^int^/CD11b^hi^ population. Renal parenchymal cells (bottom) were detected with a second CD45-specific mAb and tissue resident helper T cells (T_RMs_) were identified as the parenchymal CD45^hi^/CD4^+^/CD69^+^ population. Histogram overlays show the MFI of BV421-stained-nanobodies bound to P2X7 on microglia and T_RMs_ 4 h after i.v. injection of 15 mg/kg bivalent HLE nanobodies, controls from non-injected mice. Data are representative of three independent experiments. Dot plots shown are from a single mouse. An independent experiment refers to an experiment with a single mouse per condition, in which all mice were analyzed on the same day.

To assess the ability of P2X7-specific nanobodies to reach immune cells in various tissues following different routes of administration, we designed a flow cytometric assay that allows detection of the injected nanobody on the cell surface of P2X7-expressing cells (Figure 2A). Briefly, purified nanobodies were injected into mice either i.v. or i.c.v. or produced endogenously after intramuscular injection of nanobody-encoding AAV. Respectively after 4 h, 18 h or 120 days of injection, we evaluated the binding of P2X7-specific nanobodies on target cells. Three minutes before sacrifice, mice were injected with a PerCP-conjugated CD45-specific mAb to label vascular immune cells and thereby distinguish them from resident cells in the parenchyma (Anderson et al., 2014). Blood samples were collected to determine the concentration of unbound soluble nanobodies in serum. Residual vascular nanobodies were removed by perfusion with PBS-heparin (Figure 2A). Cell suspensions from brain and kidney were prepared and counterstained *ex vivo* with two panels of antibodies, including a second, fluorochrome-conjugated CD45-specific mAb (Figure 2B). The degree of P2X7 occupancy achieved by the injected nanobody was analyzed on brain microglia and resident memory T cells (T_RM_) from the kidney, two cell types known to express high levels of P2X7 (Stark et al., 2018; Borges da Silva et al., 2020) (Figure 2B). To validate this assay, we first injected i.v. 15 mg/kg of 1c81-dim HLE and analyzed the samples 4 h later. Bivalent HLE nanobodies bound to P2X7 on microglia and T_RMs_ were detected with anti-VHH mAbH0077, that binds to Alb11, followed by a BV421-conjugated mouse IgG1-specific mAb. For comparison, we determined the maximal occupancy of P2X7 by bivalent HLE nanobodies on both cell-types by incubating a separate aliquot with a saturating dose of the same nanobody construct (Figures 2A,B). The data validated the assay and demonstrated the presence of bivalent HLE nanobodies at the surface of both cell types, fully occupying P2X7 on kidney T_RMs_ and, as expected, at a lower level of occupancy on microglia (Figures 2A,B). We analyzed brain levels of bivalent HLE nanobodies for up to 7 days after i.v. injection and 28 days after i.c.v. injection. The time points shown in the following three figures correspond to those at which plateau levels of maximal P2X7 occupancy were reached in the brain.

### 3.3 Intravenous injection of a high dose of bivalent HLE nanobodies results in full occupancy and full blockade of P2X7 on microglia

**FIGURE 3:**
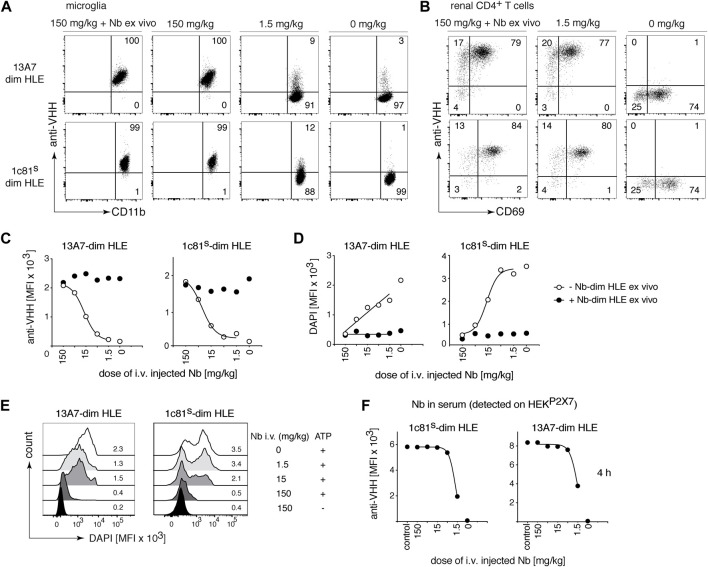
Full occupancy and full blockade of P2X7 on microglia is achieved by a high dose of intravenously injected P2X7 nanobodies. Titrated amounts of the indicated bivalent HLE nanobodies were injected i. v. and 4 h later the occupancy of P2X7 by the nanobody was analyzed on microglia and kidney-resident helper T cells (T_RMs_), as described in Figure 2. Flow cytometry dot plots of microglia **(A)** or renal CD4^+^ T cells **(B)** illustrate the relative occupancy of P2X7 achieved by the indicated concentrations of i. v. injected nanobodies. The maximal target occupancy is depicted after *ex vivo* addition of a saturating dose of nanobodies (+ Nb *ex vivo*). **(C)** Graphs display the dose-dependent MFI of P2X7 staining on microglia obtained by i. v. injection of nanobodies (open circles); closed circles = maximal staining obtained after *ex vivo* addition of saturating nanobodies. **(D)** P2X7 function, as determined by the ATP-induced DAPI uptake by microglia, was assessed by flow cytometry in the absence (open circles) or presence (closed circles) of saturating nanobodies added *ex vivo*. Cells were treated with 0.5 mM ATP at 37°C for 5 min **(E)** MFI of DAPI is displayed here as overlaid histograms. **(F)** Circulating nanobodies were analyzed in the serum of injected mice. Serum (1:100) was incubated with HEK^P2X7^ and bound bivalent HLE-nanobodies were detected by anti-VHH mAb. Graphs illustrate the MFI of HEK^P2X7^ cells versus the dose of i. v. injected Nb-dim HLE. Data are representative of three (13A7-dim HLE) and five (1c81^s^-dim HLE) **(A–C)**, or three (13A7-dim HLE) and six (1c81^s^-dim HLE) **(D–F)** independent experiments. Dot plots shown are from a single mouse. An independent experiment refers to an experiment with a single mouse per condition, in which all mice were analyzed on the same day.

Conventional antibodies (150 kDa), hardly penetrate the brain. In fact, only between 0.1 and 0.7% of the injected antibody dose was detected in the brain (Rubenstein et al., 2003; Abuqayyas and Balthasar, 2013; Bousquet and Janin, 2014; Bousquet et al., 2016). In case of nanobodies, it has been reported that a high pI increases brain penetration (Li et al., 2012; Li et al., 2016). To test whether the pI effect also modulates the BBB crossing of bivalent HLE nanobodies, mice were administered i.v. with P2X7 nanobodies with a high pI (1c81^s^-dim HLE) or a low pI (13A7-dim HLE) in serially titrated doses from 150 mg/kg to 1.5 mg/kg. Four hours later, nanobody binding to P2X7 on microglia in the brain (Figure 3A) and T_RMs_ in the kidney (Figure 3B) was analyzed. Both nanobodies bound to cell-surface P2X7 on microglia in a dose dependent manner (Figures 3A,C), reaching full occupancy of P2X7 on microglia only with the highest dose (150 mg/kg) (Figures 3A,C). Following injection of the lowest dose of 1.5 mg/kg, bivalent HLE nanobodies were barely detectable on microglia. In contrast, this low dose was sufficient to fully occupy P2X7 on T_RM_ cells within 4 h after injection (Figure 3B). These results show that the pI of a bivalent HLE nanobody does not appear to be a critical determinant for brain penetration, and that penetration into the brain parenchyma requires >100-fold higher doses of i.v. injected nanobodies than those required to penetrate into the kidney parenchyma.

To evaluate the capacity of the in vivo-bound bivalent HLE nanobodies to block gating of P2X7 on microglia, brain cells were incubated with ATP in the presence of DAPI and the ATP-induced uptake of DAPI by microglia was measured by flow cytometry (Figure 3D). The results show a dose dependent inhibition of ATP-induced DAPI uptake by microglia (Figures 3D,E). Only the highest injected dose (150 mg/kg) achieved almost complete blockade of P2X7-mediated DAPI uptake (Figures 3D,E).

The serum levels of bivalent HLE nanobodies 4 h after i.v. administration was determined by flow cytometry assay on stable HEK-mouse P2X7 cells (HEK^P2X7^) (Figure 3F). The results revealed that the level of unbound nanobodies in the serum of mice that had received doses higher than 5 mg/kg saturated all available P2X7 on HEK^P2X7^ cells, whereas the serum of mice that had received 1.5 mg/kg showed ∼2-fold lower levels of unbound nanobodies (Figure 3F). ELISA analyses (Supplementary Figure S1A) indicate that the concentration of unbound bivalent HLE nanobodies circulating in serum at 4 h correlate with the injected dose (Supplementary Figure S1B), revealing that approximately 60% of the injected dose was still circulating in serum 4 h after injection.

### 3.4 Intracerebroventricular injection of a low dose of bivalent HLE nanobodies results in full occupancy and full blockade of P2X7 on microglia

**FIGURE 4:**
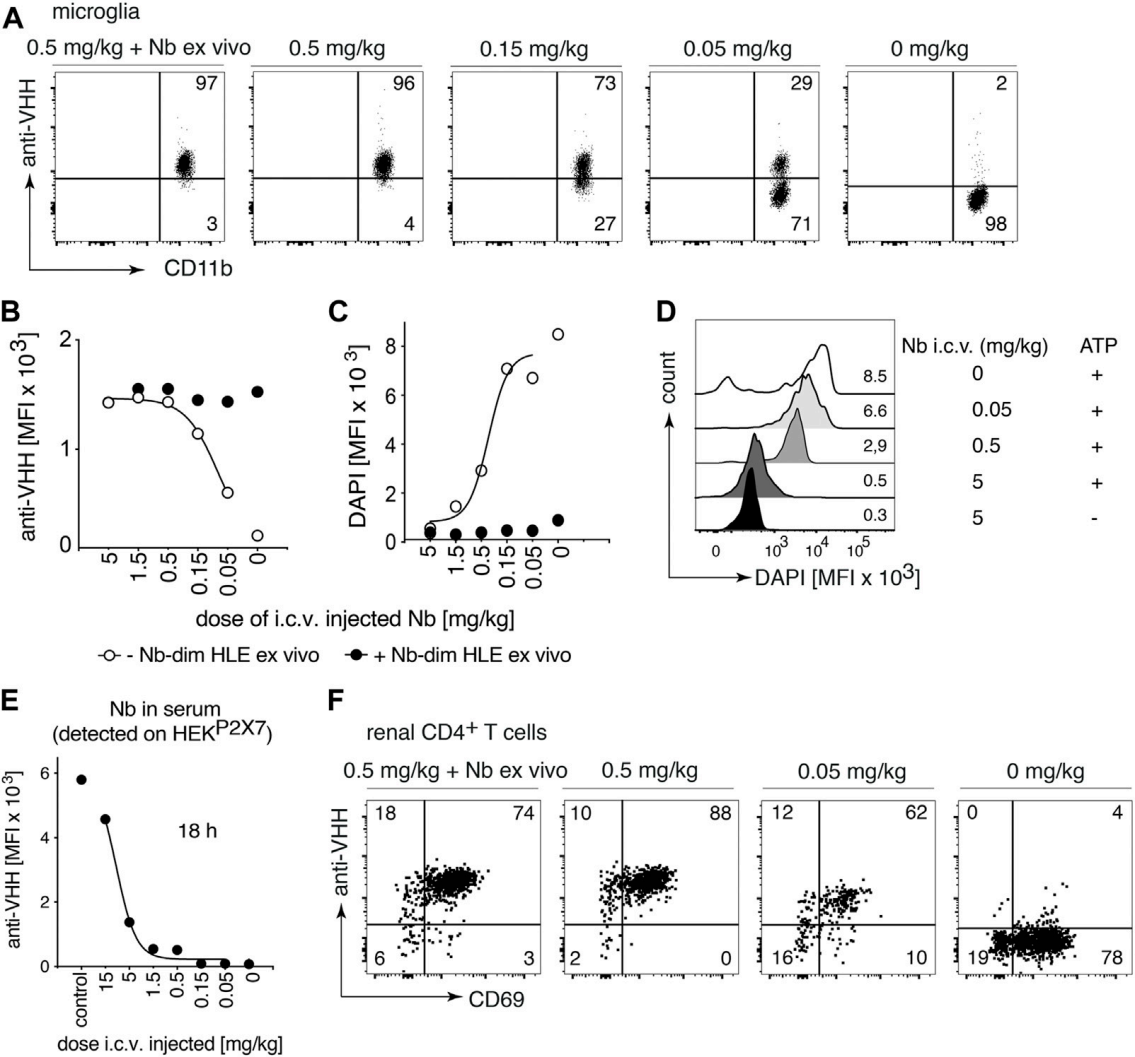
Full occupancy of P2X7 on microglia is achieved after an i.c.v. injection of a low dose of P2X7 nanobody. Titrated amounts of bivalent HLE nanobody 1c81^s^-dim HLE were injected i. c.v. and 18 h later the occupancy of P2X7 by the nanobody was analyzed on microglia and kidney-resident helper T cells (T_RMs_), as described in Figure 2. Flow cytometry dot plots of microglia **(A)** or renal CD4^+^ T cells **(F)** illustrate the relative occupancy of P2X7 achieved by the indicated concentrations of i. c.v. injected nanobodies versus the maximal occupancy achieved after *ex vivo* addition of saturating nanobodies (+ Nb *ex vivo*). **(B)** Graph displays the dose-dependent MFI of P2X7 staining on microglia obtained by i.c.v. injection of nanobodies (open circles); closed circles = maximal staining obtained after *ex vivo* addition of saturating nanobodies. **(C)** P2X7 function, as determined by the ATP-induced uptake of DAPI by microglia, was assessed by flow cytometry in the absence (open circles) or presence (closed circles) of saturating nanobodies added *ex vivo*. **(D)** MFI of DAPI displayed as overlaid histograms. **(E)** The presence of circulating nanobodies was evaluated in the serum of injected mice. Bivalent HLE-nanobodies in serum (1:100) bound to HEK^P2X7^ cells were detected by anti-VHH mAb. Graphs illustrate the MFI of HEK^P2X7^ cells versus the dose of i.c.v. injected Nb-dim HLE. Data are representative of four **(A,B and F)** or five independent experiments **(C–E)**. Dot plots shown are from a single mouse. An independent experiment refers to an experiment with a single mouse per condition, in which all mice were analyzed on the same day.

The BBB restricts the access of i.v. administered nanobodies to cells within the CNS. It has been shown that the i.c.v route of administration was efficient to deliver nanobodies to their target throughout the brain (Gomes et al., 2018). To evaluate whether P2X7 nanobodies bind to their target on microglia following i.c.v. injection, we carried out a dose response analysis with 1c81^s^-dim HLE (Figure 4). Doses from 5 mg/kg to 0,05 mg/kg were injected in a small volume (2 µl) into a cerebral ventricle and 18 h later, binding of nanobodies to P2X7 on microglia was analyzed. The results show that a dose of 0.5 mg/kg of bivalent HLE nanobodies achieved full occupancy of P2X7 on the surface of microglia, and even the low dose of 0.15 mg/kg results in almost complete P2X7 occupancy on microglia (Figures 4A,B). A dose of 5 mg/kg sufficed to fully block ATP-induced DAPI uptake by microglia (Figures 4C,D).

To determine whether i.c.v. injected bivalent HLE nanobodies had leaked into peripheral circulation, the level of nanobodies in serum was determined indirectly by staining HEK^P2X7^ cells with these sera. The results show detectable nanobody levels in circulation which, however, do not suffice to fully occupy P2X7 on HEK^P2X7^ cells (Figure 4E). The determination of the concentration of circulating bivalent HLE nanobodies by ELISA (Supplementary Figure S1A) showed that approximately 10–30% of the injected dose was detected in serum 18 h after i.c.v. injection (Supplementary Figure S1C).

To determine whether nanobodies that had leaked into peripheral circulation also reached P2X7 on the cell surface of parenchymal T cells of the kidney, renal T_RM_ cells were analyzed for occupancy of P2X7 18 h after i.c.v. administration of 1c81^s^-dim HLE (Figure 4F). The results show full occupancy of P2X7 on T_RM_ cells of the kidney after injection of doses starting at 0.5 mg/kg and higher, and a substantial occupancy already after an injection of 0.05 mg/kg (Figure 4F).

### 3.5 P2X7 nanobodies are detected on the cell surface of microglia on brain sections 24 h after i.c.v. administration

**FIGURE 5:**
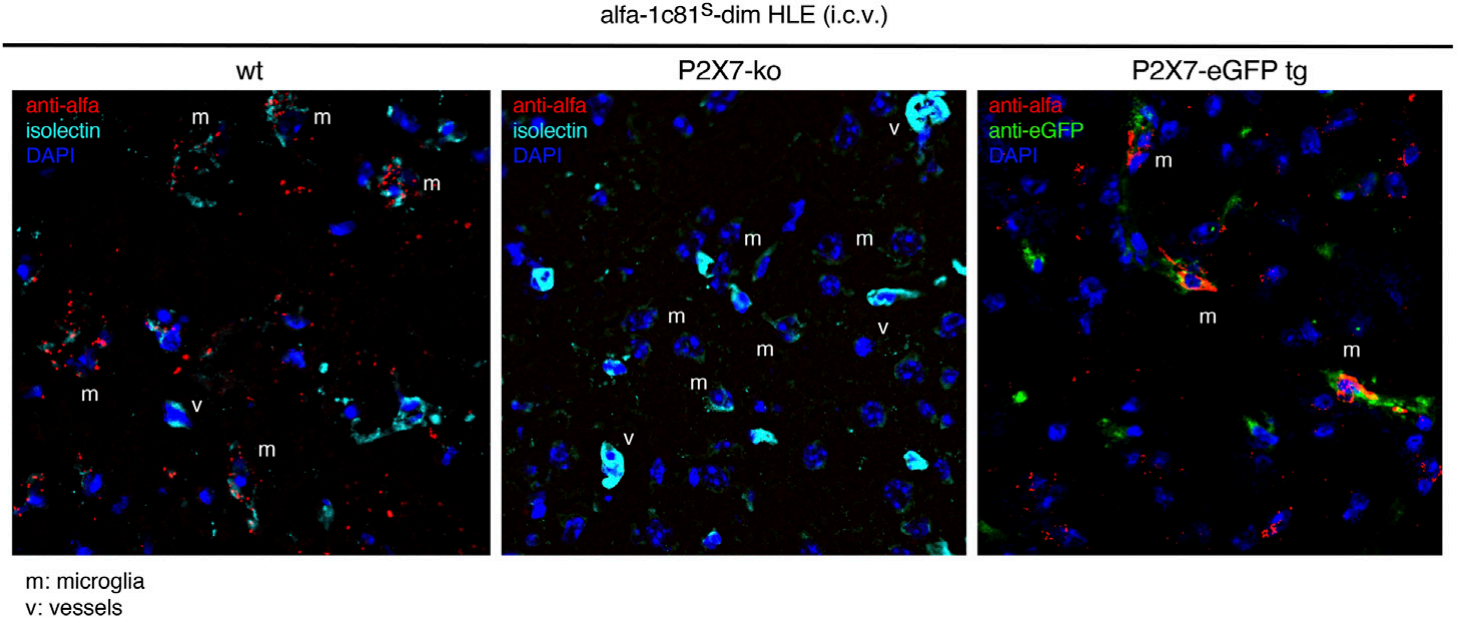
Detection of P2X7 nanobodies bound to the cell surface of microglia on brain sections. Target binding of 1c81^s^-dim HLE nanobody on brain cryosections was determined 24 h following i. c.v. injection of a low dose of 0.5 mg/kg in wildtype mice. P2X7-deficient mouse and a P2X7-eGFP transgenic mouse expressing high levels of P2X7-eGFP fusion protein were used as controls. For this experiment an ALFA-tagged version of 1c81^s^-dim HLE nanobody has been used, allowing detection on brain sections with an ALFA-specific nanobody (red). Microglia are stained with isolectin (cyan) and P2X7-eGFP with an eGFP-specific mouse monoclonal antibody (mAb) (green). (v) point to blood vessels brightly stained with isolectin and (m) shows microglial cells weakly stained with isolectin. Data are representative of two independent experiments. Dot plots shown are from a single mouse. An independent experiment refers to an experiment with a single mouse per condition, in which all mice were analyzed on the same day.

We next set out to directly visualize on brain sections the occupancy of P2X7 on the cell surface of microglia after i.c.v. injection of bivalent HLE nanobodies. Since the mAbH0077-based detection system did not yield signals in fluorescence microscopy, we constructed a bivalent HLE nanobody bearing at the N-terminus the ALFA-tag (Gotzke et al., 2019). The ALFA-tagged 1c81^s^-dim HLE nanobody was injected i.c.v. to mice (1.5 mg/kg), and 24 h later P2X7-bound nanobodies were detected *via* their ALFA-tag with an ALFA-specific nanobody. As controls we injected N-ter tagged 1c81-dim^s^ HLE into P2X7-deficient mice (Labasi et al., 2002) and P2X7-transgenic mice that overexpress a P2X7-eGFP fusion protein under the control of the endogenous P2X7 promotor (Kaczmarek-Hajek et al., 2018) (Figure 5). Our findings show that 1c81^s^-dim HLE bound to the cell surface of microglia on brain cryosections of wt mice but not that of P2X7^−/−^ mice (Figure 5). Moreover, sections of P2X7-eGFP transgenic mice showed colocalization of P2X7-eGFP with the injected nanobody on microglia (Figure 5). These results confirm that the i.c.v. injection route efficiently delivers P2X7-targeting nanobodies to microglia.

### 3.6 A low dose of i.c.v. injected bivalent HLE nanobodies suffices to rapidly and durably occupy and block P2X7 on microglia

**FIGURE 6:**
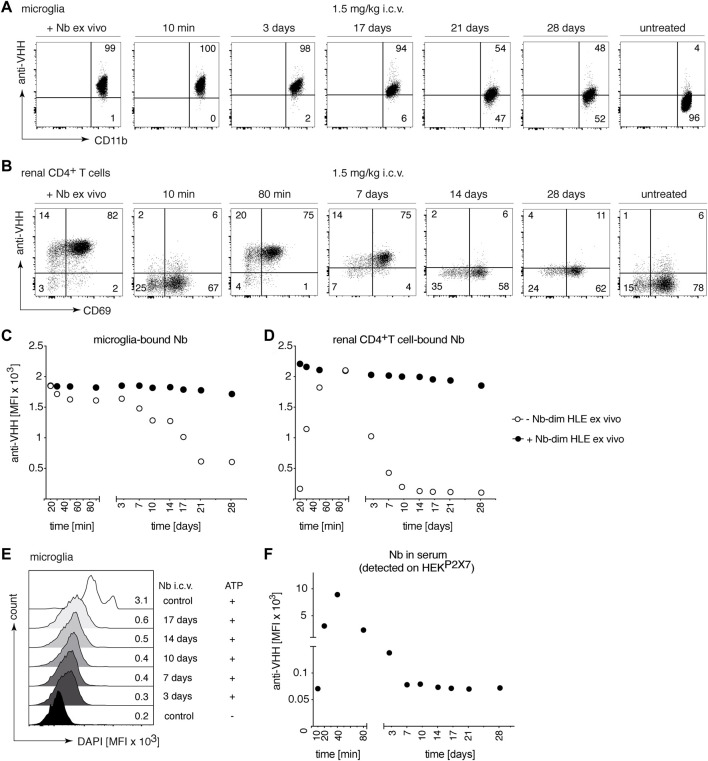
Long-term occupancy and blocking of P2X7 on microglia are achieved after a single i.c.v. injection of a low dose of P2X7 nanobody. Mice were injected i.c.v. with 1.5 mg/kg 1c81^s^-dim HLE in 2 μl volume. At the indicated time points, the occupancy of P2X7 by the nanobody was analyzed on microglia and kidney-resident helper T cells (T_RMs_), as described in Figure 2. Flow cytometry dot plots of microglia **(A)** or renal CD4^+^ T cells **(B)** illustrate the relative occupancy of P2X7 achieved at the indicated time points after i.c.v. injection of nanobodies versus the maximal occupancy achieved after *ex vivo* addition of saturating nanobodies (+ Nb *ex vivo*). Graphs display the time-dependent MFI of P2X7 staining on microglia **(C)** and renal CD4^+^ T cells **(D)** obtained by i.c.v. injection of nanobodies (open circles); closed circles = maximal staining obtained after *ex vivo* addition of saturating nanobodies. **(E)** P2X7 function, as determined by the ATP-induced DAPI uptake by microglia, was assessed by flow cytometry. Histograms display the MFI of DAPI uptake by microglia. **(F)** The presence of circulating nanobodies was evaluated in the serum of injected mice. Bivalent HLE nanobodies in serum (1:100) bound to HEK^P2X7^ cells were detected by anti-VHH mAb. Graph illustrates the MFI of HEK^P2X7^ cells versus the time after i.c.v. injection of Nb-dim HLE. Data are representative of five **(A–D and F)** or four independent experiments **(E)**. Dot plots shown are from a single mouse. An independent experiment refers to an experiment with a single mouse per condition, in which all mice were analyzed on the same day.

To study the kinetics of P2X7 occupancy on microglia by 1c81^s^-dim HLE after i.c.v. administration, we injected i.c.v. into mice 1.5 mg/kg of this nanobody in 2 μl, and analyzed P2X7 occupancy by flow cytometry at different time points from 10 min to 28 days (Figure 6). The results show, that 1c81^s^-dim HLE already completely occupied all available P2X7 on microglia within 10 min after injection (Figures 6A,C). P2X7 on microglia remained fully occupied by 1c81^s^-dim HLE until day 3. A steady decrease in the occupancy of microglial P2X7 by 1c81-dim^s^ HLE was observed in the following 2 weeks until it reached a plateau at day 21 (Figures 6A,C). Because we had previously observed that i.c.v. injected 1c81^s^-dim HLE leaked into systemic circulation and reached P2X7 on the cell surface of T_RM_ cells in the kidney (Figure 4F), we analyzed P2X7 occupancy by the injected nanobody on these cells at the indicated time points. The results show that little if any 1c81^s^-dim HLE had reached P2X7 on the cell surface of kidney T_RMs_ within 10 min after i.c.v. injection (Figures 6B,D). However, within the next hour, 1c81^s^-dim HLE leaked into peripheral circulation, reaching full occupancy of P2X7 on T_RM_ cells at 80 min after i.c.v. injection (Figures 6B,D). Subsequently, occupancy of P2X7 on T_RMs_ declined much faster than that of P2X7 on microglia (Figures 6C,D). By day 14, P2X7 occupancy on T_RMs_ had already diminished to a low level suggesting that most of the nanobody had been cleared from the circulation (Figures 6B,D). Controls showing the *ex vivo* staining with saturating amounts of nanobodies, indicate that the levels of cell surface P2X7 remained high during the entire duration of the experiment (Figures 6C,D).

Our findings demonstrated that a single i.c.v. injection of 30 μg 1c81^s^-dim HLE leads to rapid and long-term occupancy of P2X7 on microglia (Figures 6A,C). Consistently, a long-term blockade of ATP-induced DAPI-uptake by microglia was observed up to 17 days after i.c.v. injection (Figure 6E).

In addition, the level of 1c81^s^-dim HLE in serum was determined by staining of HEK^P2X7^ with sera of the injected mice (Figure 6F). The results confirmed that nanobodies were barely detectable in serum at 10 min after i.c.v. injection, but subsequently the nanobodies leaked into systemic circulation reaching maximum levels in serum at 40 min after i.c.v. injection. After this time point, the amount of 1c81^s^-dim HLE in serum progressively decreased until it was no longer detectable after day 10 (Figure 6F). Notably, the levels of 1c81^s^-dim HLE detected in serum show a similar time course of appearance and clearance as the levels of P2X7 occupancy on renal T_RM_ cells, with nanobody levels in serum peaking a few minutes earlier than on T_RMs_ (Figures 6D,F).

### 3.7 Intracerebral injection of an AAV1 encoding 13A7-dim Fc results, 28 days later, in a complete functional blockade of P2X7 on microglia and splenic T cells

**FIGURE 7:**
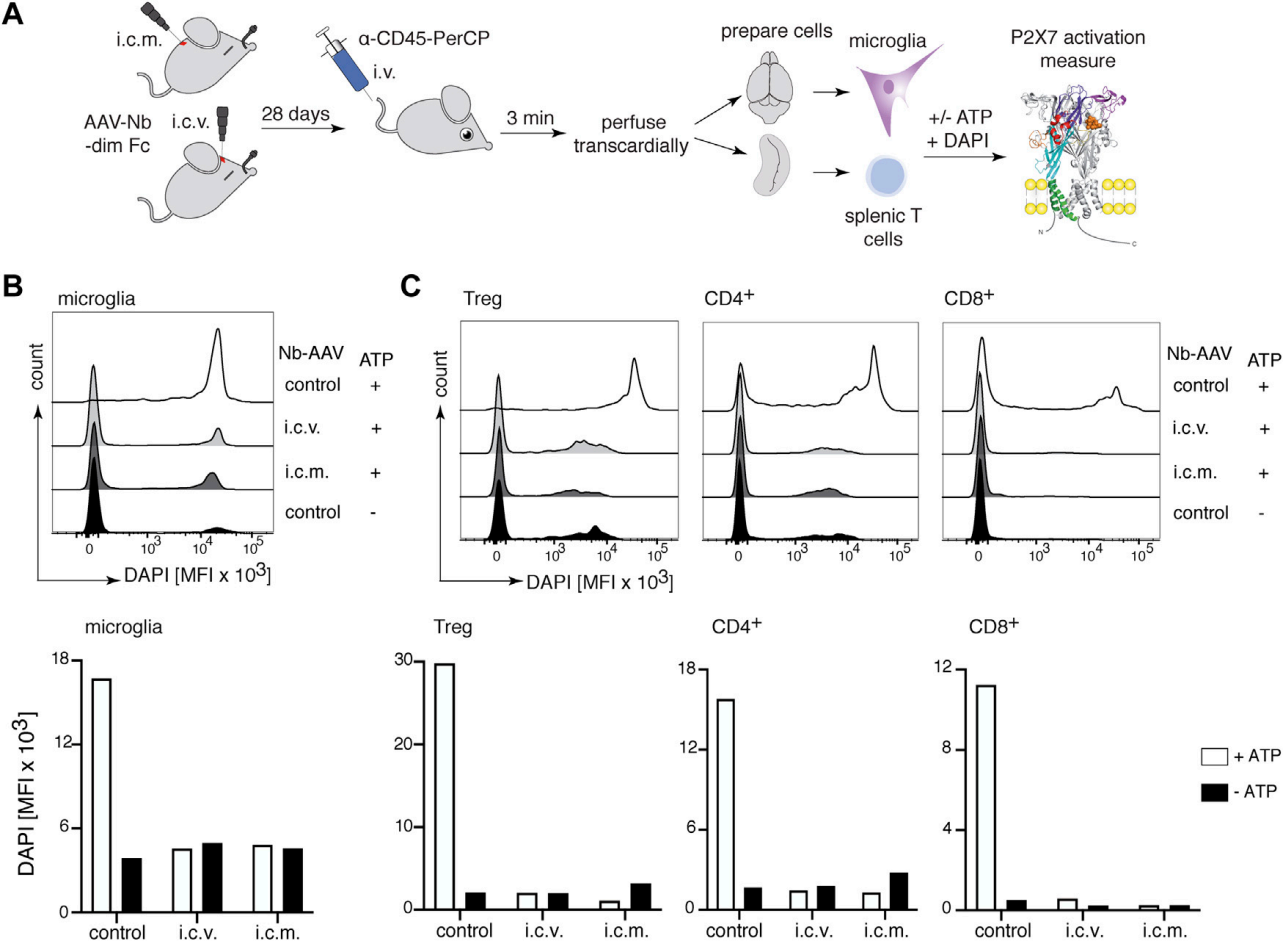
Complete inhibition of P2X7 on microglia and splenic T cells 28 days after intracerebral injection of P2X7 nanobody-encoding AAV1. **(A)** Mice were injected into the brain either i.c.v. or intracisterna magna (i.c.m.) with 10^11^ viral genomes of an AAV1 vector coding for 13A7-dim Fc (Figure 1). After 28 days, P2X7 function, as determined by the ATP-induced DAPI uptake by microglia and splenic T cells, was analyzed by flow cytometry. Histograms show the MFI of DAPI uptake by microglia **(B)** or splenic Tregs and conventional CD4^+^ and CD8^+^ T cells **(C)**. Below, bar diagrams illustrate the ATP-induced DAPI uptake by the indicated cell population of AAV1-injected mice or control mice. Data are representative of two independent experiments. Dot plots shown are from a single mouse. An independent experiment refers to an experiment with a single mouse per condition, in which all mice were analyzed on the same day.

We have recently shown that AAV-mediated *in vivo* delivery of nanobody-encoding genes is an effective alternative route for nanobody administration, i.e., by endogenous production of nanobodies by AAV transduced muscle cells (Gonde et al., 2021). To determine whether nanobodies endogenously produced in the brain can reach P2X7 on the surface of microglia, we injected i.c.v or intracisterna magna (i.c.m.) an AAV1 vector coding for the P2X7-blocking 13A7-dim Fc (Figure 1A) (Gonde et al., 2021). P2X7 blockade of DAPI-uptake was evaluated on microglia 28 days after injection of AAV1 coding for 13A7-dim Fc (Figure 7A). The results show a marked ATP-induced uptake of DAPI by microglia of control animals but not of mice injected with AAV1 coding for 13A7-dim Fc (Figure 7B). Similar effects were observed for splenic T cells (Figure 7C), indicating that nanobodies that were produced in the brain had also entered the circulation and reached the spleen. These results reveal the ability of the AAV1 to transduce cells within the CNS, which mediated the *in vivo* production and delivery of nanobodies both locally to microglia in the CNS and systemically to T cells in the spleen.

### 3.8 Complete P2X7 inhibition on microglia was achieved 120 days after i.m. injection of AAV1-encoding 13A7-dim Fc, and almost full P2X7 occupancy after injection of AAV1-encoding 13A7-dim HLE

**FIGURE 8:**
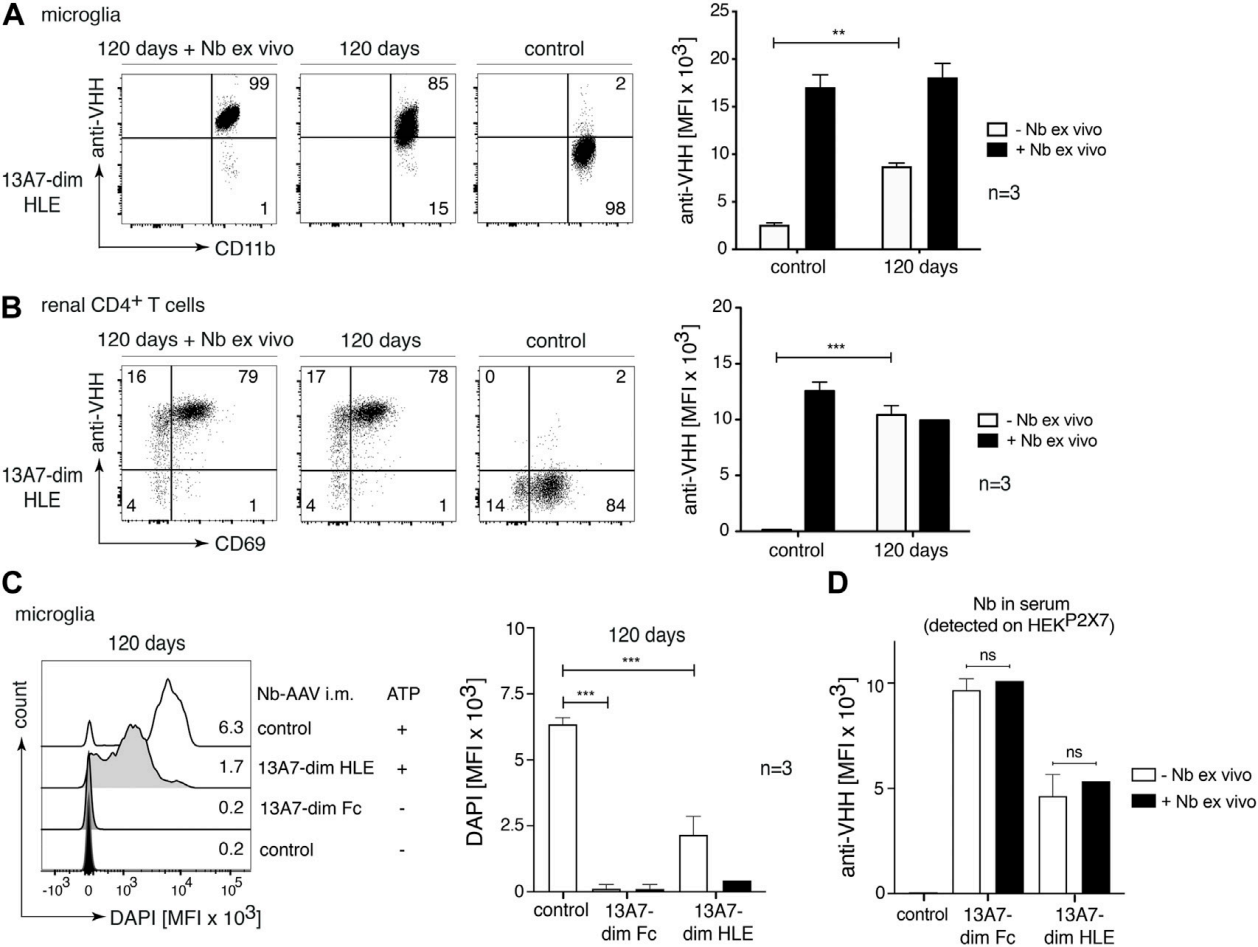
Near complete target occupancy and complete P2X7 inhibition on microglia 120 days after i.m. injection of AAV1 vectors coding for P2X7 nanobodies. Mice were injected i. m. with 10^11^ viral genomes of AAV1 coding for 13A7-dim HLE and 13A7-dim Fc. After 120 days, the occupancy of P2X7 by the nanobodies was analysed on microglia and kidney-resident helper T cells (T_RMs_), as described in Figure 2. Flow cytometry dot plots of microglia **(A)** or renal CD4^+^ T cells **(B)** illustrate the relative occupancy of P2X7 at 120 days after i.m. injection of AAV1-Nb versus the maximal occupancy achieved after *ex vivo* addition of saturating nanobodies (+ Nb *ex vivo*). Bar diagrams illustrate the occupancy of P2X7 on microglia **(A)** and renal CD4^+^ T cells **(B)** obtained from AAV1-Nb injected and control mice (n = 3/group). **(C)** P2X7 function, as determined by the ATP-induced uptake of DAPI by microglia, was assessed by flow cytometry. Bar diagrams illustrate DAPI uptake by microglia from three AAV1-Nb injected and three control mice in the absence (- Nb *ex vivo*) or presence (+ Nb *ex vivo*) of saturating nanobodies added *ex vivo*. **(D)** Circulating nanobodies were measured in the serum of transduced mice. Serum (1:100) was incubated with HEK^P2X7^ cells. Bound 13A7-dim HLE nanobodies were detected by anti-VHH mAb and BV421-conjugated IgG1-specific mAb, and bound 13A7-dim Fc nanobodies by BV421-conjugated IgG1-specific mAb. Bar diagrams illustrate the MFI of HEK^P2X7^ cells from AAV1-Nb injected and control mice (n = 3/group). Dot plots shown are from a single mouse. An independent experiment refers to an experiment with a single mouse per condition, in which all mice were analyzed on the same day.

Since our results indicate that a very high systemic dose of nanobodies (150 mg/kg) is required to detect effective brain uptake, we next sought to determine the effect of long-term endogenous production of nanobodies by AAV-transduced muscle cells. We injected into the gastrocnemius muscle AAV1 coding for P2X7 nanobodies in two bivalent formats, 13A7-dim HLE and 13A7-dim Fc (Figure 1A). The level of P2X7 occupancy by the endogenously produced nanobody-constructs was analyzed on microglia and T_RM_ cells of the kidney 120 days after AAV-transduction (Figure 8). The results show that 13A7-dim HLE had achieved a substantial coverage of P2X7 on microglia (Figure 8A) and complete P2X7 occupancy on kidney T_RM_ cells (Figure 8B). Consistently, the sustained *in vivo* expression of 13A7-dim HLE led to a significant, albeit only partial blockade of ATP-induced DAPI-uptake by microglia. Interestingly, endogenously produced 13A7-dim Fc completely blocked P2X7 function on microglia (Figure 8C). In addition, levels of 13A7 dim-HLE in serum were analyzed indirectly by staining of HEK^P2X7^ cells with sera from AAV-transduced mice (Figure 8D) and by ELISA (Supplementary Figure S1A,D). The results indicate that the serum contained saturating amounts of antibodies even after 120 days of i.m. injection of nanobody encoding AAV. Together, AAV1-nanobody delivery in the muscle provides sufficient high systemic levels of nanobodies to reach P2X7 on the cell surface of microglia in the brain.

## 4 Discussion

In the present study we show that P2X7 expressed on the cell surface of microglia can be effectively targeted by intracerebroventricular injection of P2X7 antagonistic nanobodies, as well as by the AAV-mediated endogenous production of such nanobodies after intracerebral or intramuscular injection of nanobody-encoding AAV1.

We evaluated the brain delivery of P2X7-antagonistic nanobodies to target microglia in the brain using different routes of administration: i.v., i.c.v. and the AAV-mediated endogenous production of nanobodies after transduction of brain or muscle cells with nanobody-encoding AAVs. Two antagonistic and well characterized anti-P2X7 nanobodies were used in this study, 13A7 and 1c81, which have similar blocking potencies (Danquah et al., 2016). Bivalent formats with extended serum half-life were used, generated either by fusion to the hinge and Fc domain of mouse IgG1, or by fusion to an albumin-specific nanobody. The intravenous route is the easiest to translate to the clinic but also proved to be the least effective. Very high amounts of nanobodies were necessary to achieve coverage of P2X7 on microglia. The i.c.v. route was most effective, achieving rapid and long-term coverage of P2X7 on microglia after injection of very low doses. However, as a highly invasive procedure, this would be restricted to exceptional cases in the clinic. A single injection of nanobody encoding AAV1 into either the skeletal muscle or the cerebrospinal fluid also provided effective and long-term delivery of P2X7-blocking nanobodies to microglia in the brain. A major problem of this mode of application is the difficulty to control the levels and duration of antibody production *in vivo*. Moreover, due to the induction of a host antibody response directed against the AAV capsid, this mode of application is limited, at best, to a single injection per AAV serotype.

It is well known that the BBB restricts the passage of intravenously injected therapeutic proteins from the circulation into the brain. Preclinical studies in mice showed that only a small fraction of antibodies reaches the brain after intravenous injection (e.g., 0.7% of the injected dose of 8 mg/kg) (Abuqayyas and Balthasar, 2013). In humans treated intravenously with therapeutic antibodies trastuzumab or rituximab, the concentrations in the cerebrospinal fluid reached around 0.1–0.2% of the serum concentration (Rubenstein et al., 2003; Bousquet and Janin, 2014). In addition, it has been shown that the access to the brain of systemically administered anti-rabies nanobodies is dependent on the dose and the half-life of the nanobody construct. Thus, a high dose (75 mg/kg) of intraperitoneally injected half-life extended rabies nanobodies rescued mice from disease (Terryn et al., 2014). Our results are consistent with these studies. We showed that intravenously injected nanobodies required a very high dose (150 mg/kg) in order to effectively cover P2X7 on microglia within the brain parenchyma. Previous studies reported that nanobodies with a high pI show better translocation into the brain than the corresponding variants with a lower pI (Li et al., 2012; Li et al., 2016). However, in the case of the high pI nanobody 1c81^s^, intravenous injections did not result in an improved access to microglial cells in the brain parenchyma. A possible explanation for the discrepancy between our and previous findings could be the different sizes of the nanobody constructs that were used, i.e., monomeric nanobodies (15 kDa) (Li et al., 2012; Li et al., 2016), three times smaller than those used in our study (bivalent half-life extended nanobodies, 45 kDa). Fusion of the P2X7-blocking nanobodies to a ligand for a receptor or molecule that induce transcytosis across the BBB would be a conceivable option for improving brain uptake of systemically injected proteins, e.g., fusion to a transferrin receptor binding agent (Niewoehner et al., 2014; Haqqani et al., 2018; Wouters et al., 2020; Stocki et al., 2021).

As expected, using the intracerebroventricularly route, a 100-fold lower dose of 1c81^s^-dim HLE (0.5 mg/kg) was sufficient to achieve complete occupancy and blockade of P2X7, which lasted up to 3 weeks after injection. Notably, 10 min after i.c.v. injection, P2X7 was already completely occupied on microglial cells in the brain. Interestingly, we found that i.c.v. injected nanobodies also bound to P2X7 on peripheral parenchymal CD4^+^ T cells of the kidney, indicating that the i.c.v. injected nanobody rapidly reached the blood circulation. Renal parenchymal CD4^+^ T cells revealed complete occupancy of P2X7 by the i.c.v. injected nanobody within 80 min, which lasted up to 7 days.

Furthermore, and consistent with the observed relatively low levels of circulating nanobodies in serum, the occupancy of P2X7 by the injected nanobody faded more rapidly on parenchymal renal CD4^+^ T cells than on microglia. These findings are consistent with the notion that proteins are cleared from the CSF to blood circulation *via* arachnoid projections and lymphatic vessels (Ma et al., 2017; Hladky and Barrand, 2018).

Finally, our study evaluated the capacity of endogenously produced nanobodies to reach P2X7 on microglia. In a previous study we had shown that AAV-mediated delivery of nanobody-encoding genes into the skeletal muscle results in long term endogenous production of nanobodies by the AAV-transduced muscle cells (Gonde et al., 2021). Here we show that a single injection of a heavy chain antibody-encoding AAV1 into the intracerebral ventricle or the cisterna magna results in an efficient production of the antibodies in the brain, leading to a full functional blockade of P2X7 within at least 28 days. Moreover, even a single intramuscular injection of AAV1 coding for 2 different formats of P2X7 nanobodies (dim-Fc or dim-HLE) achieved a strong functional blockade of P2X7 on microglia 120 days after transduction. The slightly more effective blockade achieved by the heavy chain antibody (dim-Fc) in comparison to the bivalent-HLE (dim-HLE) could reflect differences in endogenous production or longer serum half-life in mice.

In conclusion, our data provide new and deeper insights into the conditions (route, dose, and time of administration) for nanobody-mediated targeting of P2X7 on microglia. Our results suggest that, in particular, the nanobody-encoded AAV delivery, either intracerebral or intramuscular, could be a promising new therapeutic strategy for the treatment of sterile brain inflammation.

## Data Availability

The original contributions presented in the study are included in the article/Supplementary Material, further inquiries can be directed to the corresponding author.

## Abbreviations

AAV, adeno-associated viral vector; i.v., intravenous; i.c.v., intracerebroventricular; i.c.m., intracisterna magna; i.m., intramuscular; Nb, nanobody; VHH, variable heavy-chain antibody fragment.
