# Supplementary material for: Effective targeting of microglial P2X7 following intracerebroventricular delivery of nanobodies and nanobody-encoding AAVs
Source: Front Pharmacol. 2022 Oct 10;13:1029236. doi: 10.3389/fphar.2022.1029236 (PMC9589454; doi:10.3389/fphar.2022.1029236)
Supplement: Supplementary file 1 [file DataSheet1.docx]

Supplementary Material

# Supplementary Figures

## Supplementary Figures

##
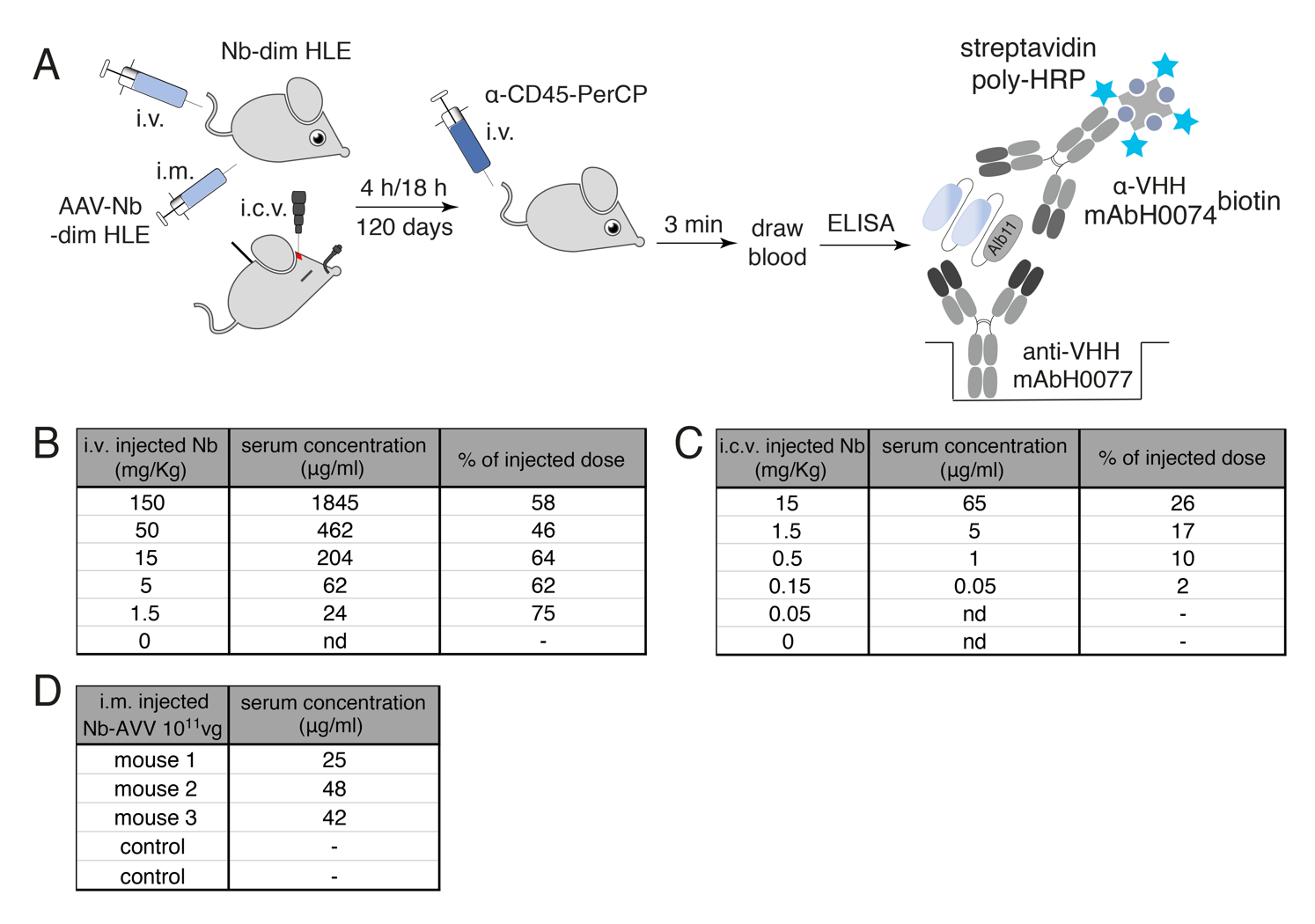


**Supplementary Figure 1.** **Quantification of P2X7 nanobodies in the serum of mice assessed by a sandwich ELISA. (A)** Schematic diagram of sandwich ELISA to assess the levels of HLE nanobodies in serum. Wells were coated with 100 ng mAbH0077. Bivalent half-life extended P2X7 nanobodies were detected with biotinylated mAbH0074 and poly-HRP-conjugated streptavidin. **(B-D)** A serial dilution of the respective bivalent HLE nanobody was used to obtain standard curves and serial dilutions of the serum were used to calculate the serum concentrations. The % of the injected dose was calculated by assuming a total serum volume of 1 ml per mouse. **(B)** The results correspond to serum obtained from mice after i.v. **(Fig. 3)** or i.c.v. injections **(Fig. 4)** of purified dim HLE nanobodies, or after i.m. injections of nanobody-encoding AAVs **(Fig. 8)**.
